# Agonist Anti-GITR Monoclonal Antibody Induces Melanoma Tumor Immunity in Mice by Altering Regulatory T Cell Stability and Intra-Tumor Accumulation

**DOI:** 10.1371/journal.pone.0010436

**Published:** 2010-05-03

**Authors:** Adam D. Cohen, David A. Schaer, Cailian Liu, Yanyun Li, Daniel Hirschhorn-Cymmerman, Soo Chong Kim, Adi Diab, Gabrielle Rizzuto, Fei Duan, Miguel A. Perales, Taha Merghoub, Alan N. Houghton, Jedd D. Wolchok

**Affiliations:** 1 Department of Medicine, Memorial Sloan-Kettering Cancer Center, New York, New York, United States of America; 2 Immunology Program, Sloan-Kettering Institute for Cancer Research, New York, New York, United States of America; 3 Weill Medical College of Cornell University, New York, New York, United States of America; New York University, United States of America

## Abstract

In vivo GITR ligation has previously been shown to augment T-cell-mediated anti-tumor immunity, yet the underlying mechanisms of this activity, particularly its in vivo effects on CD4+ foxp3+ regulatory T cells (Tregs), have not been fully elucidated. In order to translate this immunotherapeutic approach to the clinic it is important gain better understanding of its mechanism(s) of action. Utilizing the agonist anti-GITR monoclonal antibody DTA-1, we found that in vivo GITR ligation modulates regulatory T cells (Tregs) directly during induction of melanoma tumor immunity. As a monotherapy, DTA-1 induced regression of small established B16 melanoma tumors. Although DTA-1 did not alter systemic Treg frequencies nor abrogate the intrinsic suppressive activity of Tregs within the tumor-draining lymph node, intra-tumor Treg accumulation was significantly impaired. This resulted in a greater Teff:Treg ratio and enhanced tumor-specific CD8+ T-cell activity. The decreased intra-tumor Treg accumulation was due both to impaired infiltration, coupled with DTA-1-induced loss of foxp3 expression in intra-tumor Tregs. Histological analysis of B16 tumors grown in Foxp3-GFP mice showed that the majority of GFP+ cells had lost Foxp3 expression. These “unstable” Tregs were absent in IgG-treated tumors and in DTA-1 treated TDLN, demonstrating a tumor-specific effect. Impairment of Treg infiltration was lost if Tregs were GITR^−/−^, and the protective effects of DTA-1 were reduced in reconstituted RAG1^−/−^ mice if either the Treg or Teff subset were GITR-negative and absent if both were negative. Our results demonstrate that DTA-1 modulates both Teffs and Tregs during effective tumor treatment. The data suggest that DTA-1 prevents intra-tumor Treg accumulation by altering their stability, and as a result of the loss of foxp3 expression, may modify their intra-tumor suppressive capacity. These findings provide further support for the continued development of agonist anti-GITR mAbs as an immunotherapeutic strategy for cancer.

## Introduction

GITR (glucocorticoid-induced tumor necrosis factor (TNF) receptor, or TNFRSF18) is a type I transmembrane protein with homology to other TNF receptor family members such as OX40, CD27, and 4-1BB.[1] GITR is normally expressed at low levels on resting CD4+foxp3- and CD8+ T cells, but is constitutively expressed at high levels on CD4+CD25+foxp3+ regulatory T cells (Tregs). Expression increases on all 3 subpopulations following T-cell activation. GITR ligation provides a co-stimulatory signal that enhances both CD4+ and CD8+ T-cell proliferation and effector functions, particularly in the setting of suboptimal TCR stimulation.[2], [3], [4], [5] In addition, co-stimulation through GITR has been shown to make naïve or effector T cells (Teffs) resistant to the suppressive effects of Tregs in vitro, and can enhance auto-reactive, allo-reactive, and anti-viral T-cell responses in vivo.[2], [6], [7], [8], [9], [10], [11], [12], [13] This makes targeting GITR a potential immunotherapeutic approach to cancer treatment.

Recently, we and others have demonstrated that in vivo GITR ligation using an agonist anti-GITR mAb, DTA-1, can augment anti-tumor T-cell responses and induce tumor rejection in B16 melanoma and other murine models.[14], [15], [16], [17], [18], [19] However, the mechanism(s) by which GITR ligation leads to tumor rejection remain unclear. The direct co-stimulation of tumor-specific effector CD4+ and CD8+ T cells (Teffs) has been demonstrated, particularly in combination with active vaccination [16], [17], [19]; yet, the in vivo effects of DTA-1 on Tregs have not been well-defined. In fact, prior in vitro studies have suggested that the ability of DTA-1 to “block” Treg suppressive activity is due solely to its co-stimulation of Teffs, with little to no impact on Tregs themselves.[6]

In this study, we demonstrate that when used as a monotherapy, DTA-1 modulates both Tregs and Teff during treatment of B16 melanoma. In addition, GITR expression by both Teffs and Tregs was needed for the full effects of DTA-1. We show that while in vivo GITR ligation does not globally abrogate Treg suppressive activity, it does impair Treg tumor infiltration and leads to loss of foxp3 expression within intra-tumor Tregs, suggesting a localized abrogation of suppression. The net result is an augmented intra-tumor Teff:Treg ratio and greater Teff activation and function within the tumor.

## Results

### GITR expression is upregulated on tumor-infiltrating Tregs and CD8+ T cells during B16 melanoma growth

**Figure 1:**
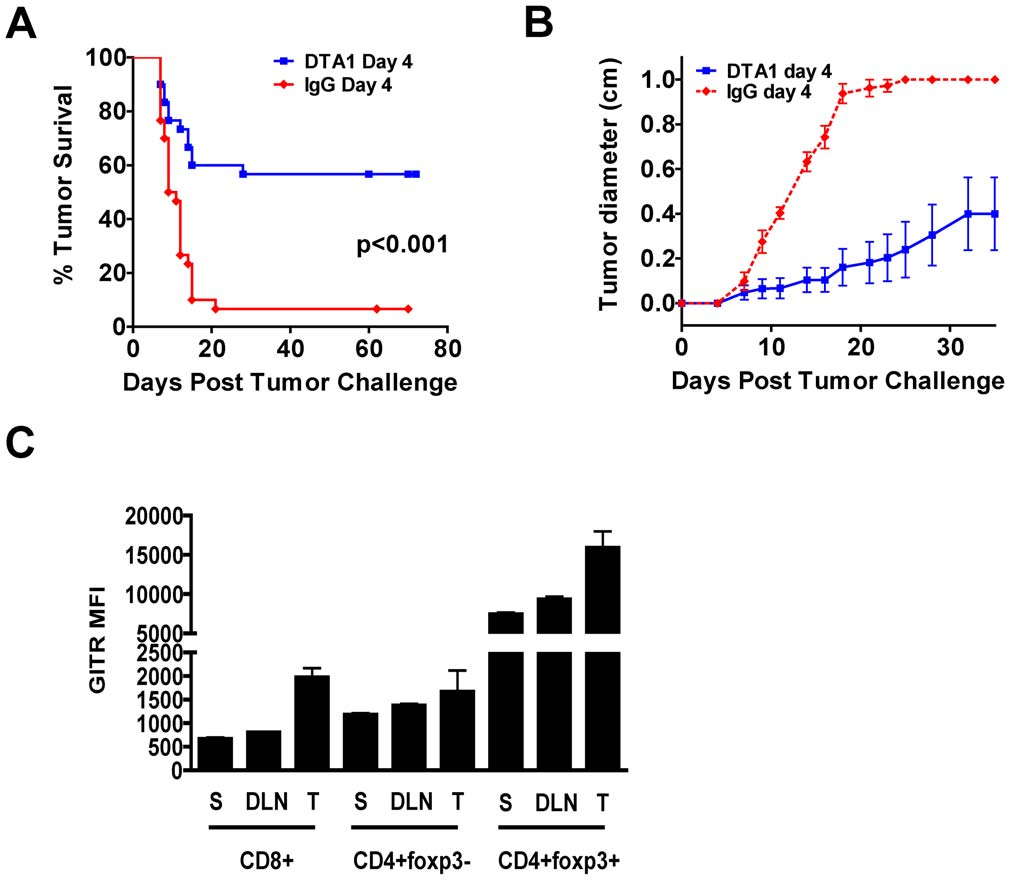
Upregulation of GITR expression correlates with optimal timing of single dose DTA-1 therapy. **A** and **B**. C57BL/6 mice (n = 10/group) were challenged intradermally with 50,000 B16 melanoma cells and treated with 1 mg DTA-1 or rat IgG i.p. on day 4 after tumor challenge. Tumor survival (A) and mean tumor diameter + SEM over time is depicted (B) **C**. Untreated mice (n = 3/group) bearing 4 day-old B16 matrigel tumors (500,000 cells) were sacrificed and lymphocytes isolated from spleens (S), tumor-draining lymph nodes (DLN), and tumors (T), were stained for CD4, CD8, foxp3, and GITR. Mean GITR fluorescence intensity (MFI) and SEM within each T cell subset is depicted.

We have shown previously that in vivo GITR ligation by DTA-1 can induce rejection of B16 melanoma tumors when administered multiple times starting 1 day after tumor challenge [18]. Although we established that DTA-1 can cure very early melanoma tumors, our prior research did not differentiate its contribution to the priming phase versus the effector phase of the immune response. Therefore, to more fully comprehend the mechanisms of GITR ligation therapy, we examined the effects of a single dose of DTA-1 at different time points post-tumor challenge to understand the consequences of ligation at distinct phases of the immune response. We found that 1 mg of DTA-1 on day 4 of tumor growth led to long-term tumor-free survival in 50–60% of C57BL/6 mice (Figures 1A, 1B). As in other tumor models [15], DTA-1 was more effective when given after several days of tumor growth, with nearly twice as many mice treated on day 4 rejecting tumors compared with mice treated on the day of tumor inoculation (Figure S1).

This suggests that GITR ligation therapy in B16 requires initiation of priming, which correlates with in vitro data showing that upregulation of GITR expression on T cells requires 48–72 hours after TCR-mediated activation [7], [13]. Thus, we hypothesized that the greater efficacy on day 4 was due, in part, to increased GITR expression on tumor-activated T cells, providing a more abundant target for ligation by the agonist antibody. In fact, we found that by day 4 after B16 inoculation, GITR expression was significantly higher on tumor-infiltrating CD8+ and Treg cells than on these populations in the spleen or tumor-draining lymph node (TDLN) (Figure 1C). This increased expression was maintained within the tumor environment on days 7 and 14 of tumor growth (data not shown). However, in contrast to the fibrosarcoma or colon carcinoma models previously reported [15], [17], as well as to our experience in an A20 lymphoma model (Cohen A, unpublished data), starting treatment with DTA-1 on day 7 or later was generally ineffective (Figure S1). This demonstrates that optimal timing of in vivo GITR ligation likely varies according to strain and the underlying immunogenicity and aggressiveness of the tumor subtype.

### In vivo GITR ligation does not systemically alter capacity of Tregs to suppress or Teffs to resist suppression

**Figure 2:**
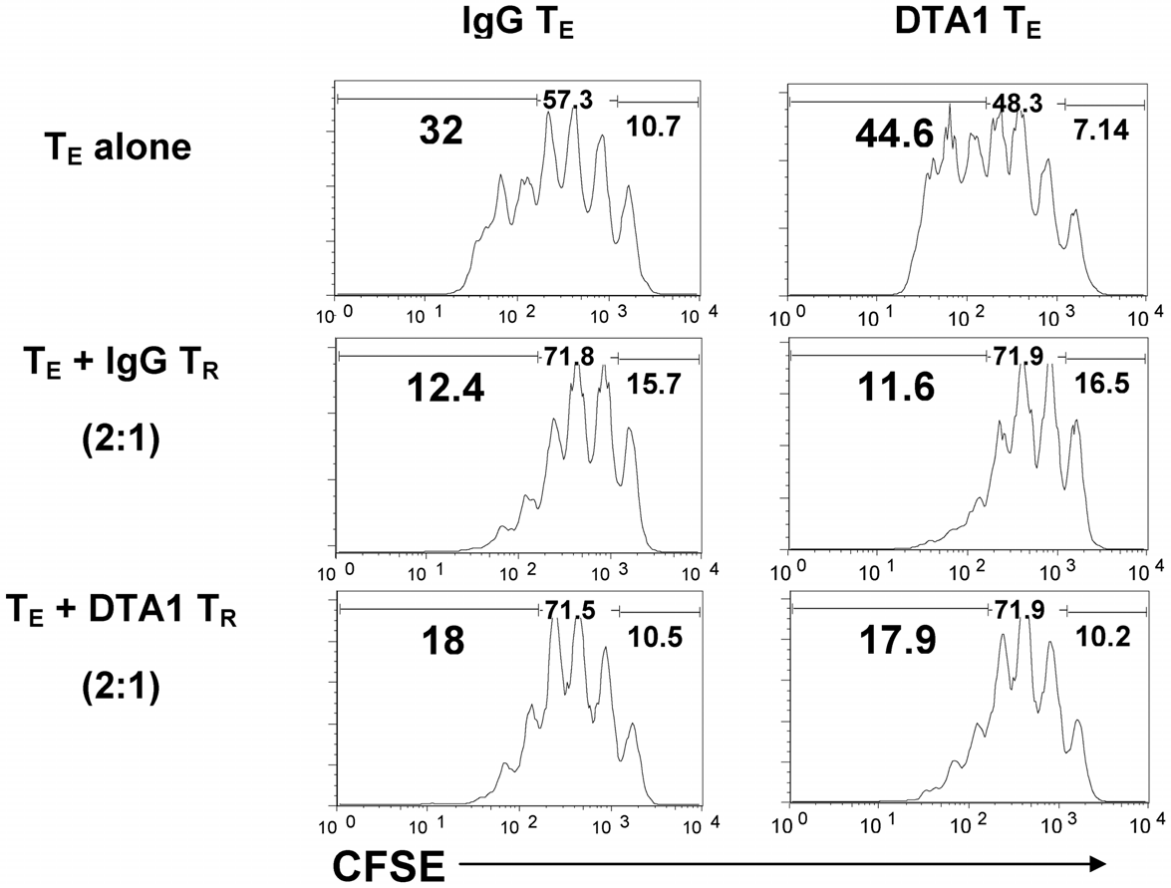
In vivo GITR ligation does not globally alter capacity of Tregs to suppress or CD8+ Teffs to resist suppression. B16-bearing C57BL/6 mice (n = 6/group) received DTA-1 or IgG on day 4. TDLN were harvested on day 7 and CD8+ (Teff) and CD4+CD25+ (Treg) cells were isolated by MACS beads. CFSE-labeled, IgG- or DTA-1-treated Teff were cultured with IgG- or DTA-1-treated Treg at 2∶1 Teff:Treg ratio, with irradiated APCs and anti-CD3 mAb 1 µg/ml for 4 days. **A**. Representative sample showing CFSE dilution in CD8+DAPI- cells.

To investigate the cell-intrinsic effects of DTA-1, we next explored how agonist anti-GITR mAb might be modulating Teff and Treg function. Highly purified Tregs were isolated from tumor-draining lymph nodes (TDLN) of B16-bearing foxp3*^GFP^* mice [20] after treatment with DTA-1 or IgG, and tested for their ability to suppress proliferation of CD8+ Teffs isolated from the same TDLN. Consistent with prior in vitro [6] and in vivo [17] studies, DTA-1-treated Tregs maintained suppressive capacity (Figure 2). In addition, neither in vitro nor in vivo GITR ligation on Tregs led to a significant change in expression of granzyme B, IL-10, or TGF-β, three molecules reported to play a role in Treg-mediated suppression in vivo [21] (Figure 2). Surprisingly, CD8+ T cells from DTA-1-treated mice remained susceptible to Treg-mediated suppression, without significant difference from those from IgG-treated mice. We observed similar findings when DTA-1 treated CD8+ T cells were co-cultured with naïve (as opposed to tumor-experienced) Tregs, again displaying no acquired resistance to ex vivo suppression (data not shown). Thus, at least within the TDLN, where initial priming of the anti-tumor effector response is likely occurring, in vivo GITR ligation does not appear to systemically alter the capacity of Tregs or CD8+ Teffs to suppress or be suppressed, respectively.

### GITR ligation leads to enhanced intra-tumor CD8:Treg ratio, and greater CD8+ effector T-cell activity within the tumor

**Figure 3:**
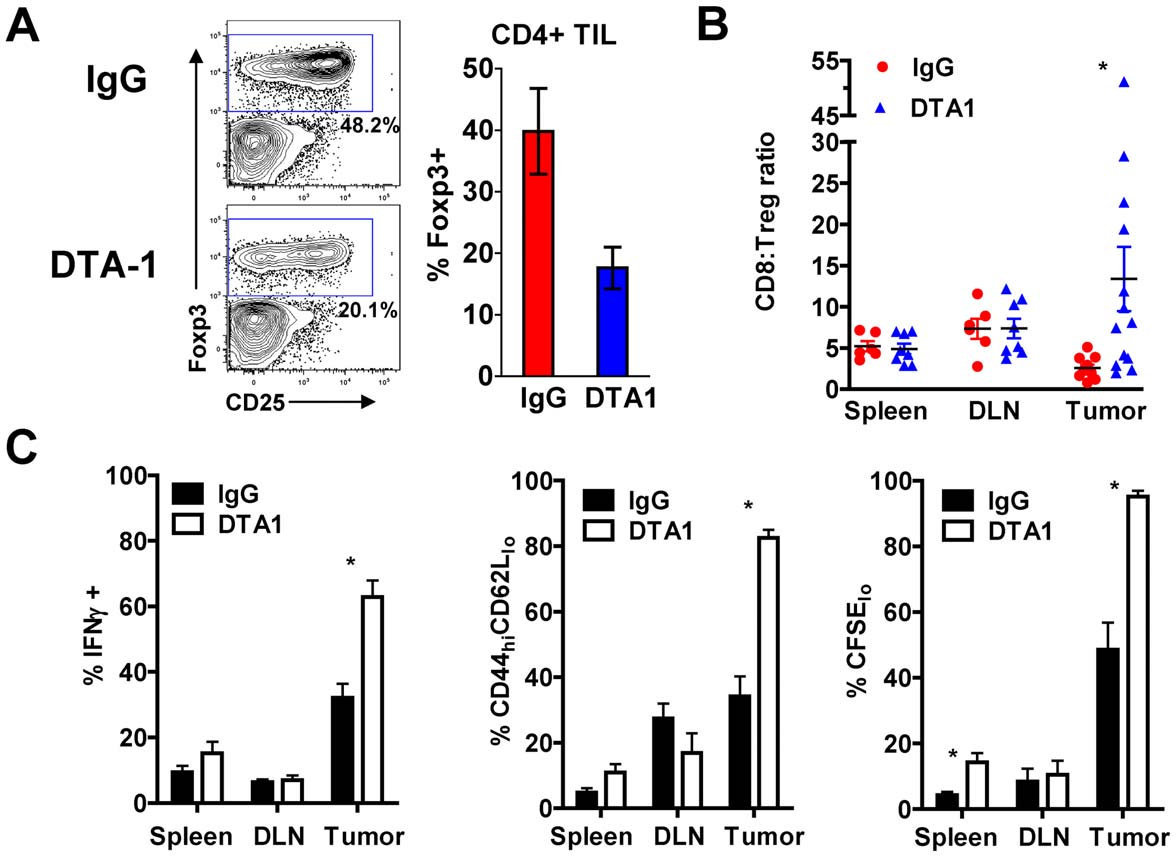
GITR ligation by DTA-1 limits Treg accumulation within the tumor and enhances intra-tumor CD8+ T-cell activity. **A**. and **B**. B16-bearing mice treated with DTA-1 or IgG on day 4 had spleens, TDLN, and tumors harvested on day 10 and lymphocytes analyzed by FACS. **A**. Representative FACS plots with gate frequencies, (left) and mean +SEM for frequency of Tregs within live CD4+ TIL gate (right) are shown. **B**. Ratio of CD8+ to CD4+foxp3+ cells in spleen, TDLN, and tumor. *p = 0.05 compared with IgG tumor. Pooled data from 3 independent experiments are shown. **C**. Naïve C57BL/6 mice (n = 3−5/group) received 4×10^6^ CFSE-labeled pmel-1 Thy1.1+CD8+ T cells 1 day prior to B16 inoculation. Recipients received DTA-1 or IgG on day 4, and donor pmel-1 CD8+ cells analyzed in spleens, TDLN, and tumors on day 14. Mean frequency +SEM is shown for IFNγ+ (**left**), activated CD44^hi^CD62L^lo^ phenotype (**center**) and proliferation by CFSE dilution (**right**) of transferred pmel-1 T cells is shown. For IFNγ recall assay (C, left), lymphocytes from spleen, TDLN, and tumor were re-stimulated for 6 hours with irradiated, gp100_25-33_ peptide-pulsed EL4 cells. Background IFNγ production for lymphocytes cultured with unpulsed EL4 cells was <1%. Over 85% of IFNγ+ cells were also CD107a+ (data not shown). * p<0.05 compared with IgG group. Representative of 3 independent experiments.

To investigate how in vivo GITR ligation modulates T cell subsets within the tumor, we first examined the relative intra-tumor frequencies of Tregs and Teffs in B16-bearing hosts following DTA-1 treatment. DTA-1 led to a significant decrease in intra-tumor Treg frequency as a percentage of CD4+ TILs (40% of CD4+ TIL for IgG, compared with 18% for DTA-1, p = 0.02, Figure 3A). This was accompanied by a modest increase in the total intra-tumor CD8:CD4 T-cell ratio (0.7 for IgG compared with 1.3 for DTA-1-treated mice, p = 0.04). The net result, therefore, was a five-fold increase in the intra-tumor CD8:Treg ratio (13.4 for DTA-1-treated compared with 2.6 for IgG-treated mice, p = 0.05), favoring the effector population (Figure 3B). Evaluation of absolute numbers/gram of tumors for Teffs and Tregs paralleled the frequency data, showing decreased Tregs without major increases in CD8+ T cell numbers/gram of tumor (Figure S3). This demonstrated that the augmented CD8:Treg ratio within tumors from DTA-1 treated mice resulted primarily from changes in the Treg compartment. No significant changes in these relative frequencies were seen in spleen or TDLN, demonstrating a specific effect within the tumor (Figure 3B). Interestingly, the proportion of mice with a >2-fold increase in intra-tumor CD8:Treg ratio over the IgG control (8/13, 61%) mirrored the percentage of mice with long-term tumor-free survival (∼60%, Figure 1A and Figure S1). This suggests that achieving a skewed CD8:Treg ratio may be predictive of long-term tumor control, as described recently with other immune-modulating antibodies.[22], [23]

We next investigated possible functional consequences of this altered intra-tumor Teff:Treg ratio by exploring the effects of GITR ligation on tumor-specific CD8+ effector cells. This was accomplished by adoptively transferring and tracking CD8+ T cells from Thy1.1+ pmel-1 TCR transgenic mice, which are specific for the melanoma antigen gp100, into C57BL/6 (Thy1.2+) mice 1 day prior to B16 challenge. GITR ligation did not consistently increase the frequency or absolute number of donor pmel-1 cells infiltrating the tumor (data not shown). However, DTA-1 significantly augmented the activation of those cells that did traffic to tumor, leading to enhanced effector function (IFN-γ secretion following gp100 peptide re-stimulation) activation (CD44^hi^CD62L^lo^ phenotype), and proliferation (as measured by CFSE dilution) (Figure 3C). IFN-γ-secreting pmel-1 T cells also mobilized CD107a (LAMP-1), a surrogate for lytic degranulation [24], demonstrating their cytolytic capacity (data not shown). These data indicate that GITR ligation results in a more effective intra-tumor CTL population, which may be a consequence of both direct CD8+ T-cell co-stimulation and a more favorable local CD8:Treg ratio.

### Treatment with agonist anti-GITR mAb modulates Treg accumulation in the tumor

**Figure 4:**
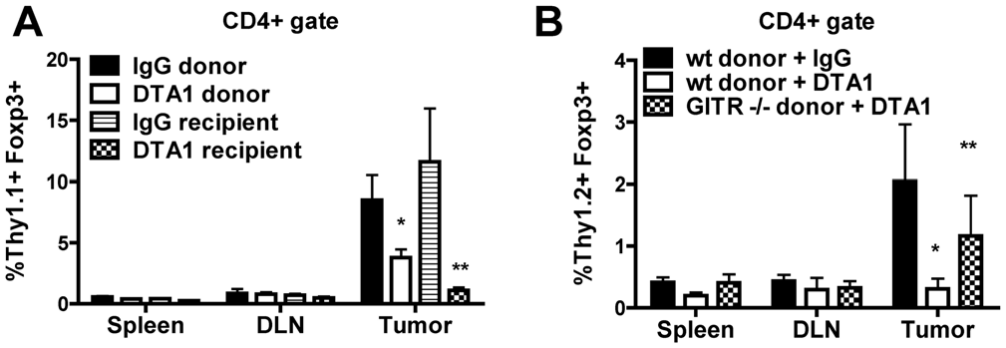
DTA-1 reduces Treg tumor trafficking in a cell intrinsic manner. **A**. B16-bearing Thy1.1+ donor mice were treated on day 5 with DTA-1 (“DTA-1 donor”) or IgG (“IgG donor”), and Tregs isolated on day 8 from their spleens and TDLN were transferred (0.7×10^6^ per recipient) to B16-bearing Thy1.2+ mice, treated 1 day earlier with cyclophosphamide 250 mg/kg i.p. Some recipients received Tregs from untreated B16-bearing donors and were then treated with DTA-1 (“DTA-1 recipient”) or IgG (“IgG recipient”) 12 hours after adoptive transfer. Recipient spleens, TDLN, and tumors were harvested 4 days after transfer. The percentage + SEM of donor (Thy1.1+CD4+foxp3+) Tregs within the total live CD4+ gate is depicted. *p = 0.05 compared with IgG donor, **p = 0.04 compared with IgG recipient. **B**. Tregs from Day 8 B16-bearing Thy1.2+ GITR^−/−^ (“KO donor”) or GITR^+/+^ (“wt donor”) donors were transferred into day 8 B16-bearing Thy1.1+ recipients treated 1 day earlier with cyclophosphamide 250 mg/kg i.p. Recipients received DTA-1 or IgG 12 hours post-transfer, and spleens, TDLN, and tumors were harvested 4 days later. The % + SEM of donor (Thy1.2+CD4+foxp3+) Tregs within the total live CD4+ gate is depicted. *p = 0.07 compared with wt donor + IgG, **p = 0.24 compared with wt donor + IgG.

Because Treg frequency was decreased only in the tumor and not systemically, we explored the hypothesis that GITR ligation was altering the migraton of Tregs to the tumor. Tumor-experienced Tregs isolated from B16-bearing, Thy1.1+ mice treated 72 hours earlier (day 4 of tumor growth) with DTA-1 or IgG were adoptively transferred into B16-bearing Thy1.2+ recipients. Recipient mice were treated 1 day earlier with cyclophosphamide to reduce endogenous lymphocyte populations and aid recovery of donor cells. While DTA-1-treated and IgG-treated Tregs distributed equally within the spleen and TDLN, there was a 50% reduction in the intra-tumor accumulation of DTA-1-treated donor Tregs, compared to IgG-treated controls (Figure 4A). The effect was even more dramatic when the recipients were treated with DTA-1 following donor Treg transfer, with a >90% reduction in donor Treg frequency within the tumor, despite equal distribution within spleen and TDLN. To determine if this effect was Treg-intrinsic, we repeated this experiment using Tregs from GITR^+/+^ or GITR^−/−^ donors. Tregs from GITR^−/−^ mice did not show a significant block in trafficking when transferred into DTA-1 treated animals, compared to IgG controls, demonstrating a requirement for direct GITR ligation on Tregs themselves (Figure 4B).

### GITR ligation leads to loss of foxp3 expression within intra-tumor Tregs

**Figure 5:**
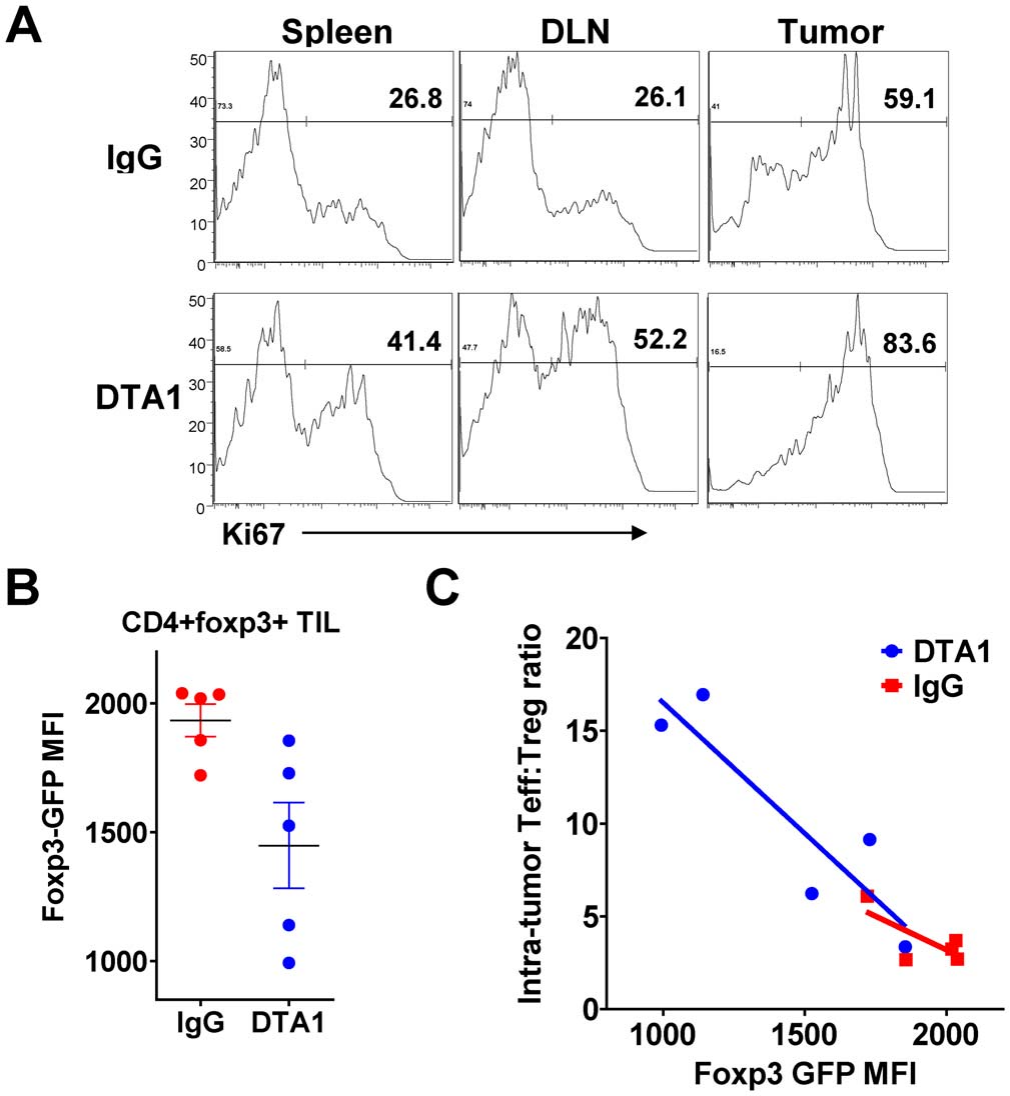
Intra-tumor Tregs show increased proliferation and lower Foxp3GFP MFI after DTA-1 treatment. **A**. Representative Ki67 expression within live CD4+foxp3+ cells isolated from day 10 B16-bearing C57BL/6 mice treated with DTA-1 or IgG on day 4. **B**. and **C**. TIL were isolated from day 10 B16-bearing *foxp3* ^GFP^ mice (n = 5/group) treated with DTA-1 or IgG on day 4 and examined by FACS. **B**. Mean fluorescence intensity (MFI) of GFP on live intra-tumor CD4+GFP+ cells. **C**. Intra-tumor CD8:Treg ratio of DTA-1- and IgG treated mouse in (**B**) is plotted against its foxp3^GFP^ MFI, demonstrating a significant inverse correlation between Teff:Treg ratio and foxp3GFP MFI in DTA-1 treated mice only (p = 0.03). Data are representative of 3 independent experiments.

The above experiments suggest that one mechanism of DTA-1's anti-tumor efficacy may be interfering with Treg infiltration into tumors, and imply a requirement for GITR expression on Tregs. However, these studies examined a small transferred population in a lympho-depleted recipient, which may not fully reflect the biologic effects of GITR ligation under steady-state conditions. We therefore examined other possible mechanisms that could contribute to the DTA-1-induced decrease in intra-tumor Tregs, including impaired proliferation, impaired survival, or loss of foxp3 expression. We found no evidence for impaired proliferation, in fact, in vivo GITR ligation on Tregs increased their proliferation in the spleen, TDLN, and tumor (Figure 5A), consistent with GITR's known co-stimulatory effects. Likewise, we could not find evidence of impaired survival following in vivo GITR signaling. Tregs within TDLN and tumors from DTA-1-treated mice showed no difference in apoptosis compared with IgG-treated controls, either by immunoflourescence (TUNEL (Figure S4A) or flow cytometric staining (Annexin V, Figure S4B, or activated caspase 3 (data not shown)) We did observe, however, that the mean fluorescence intensity (MFI) of the foxp3-GFP fusion protein was slightly lower within intra-tumor Tregs from DTA-1-treated foxp3*^GFP^* mice (Figure 5B), without changes seen in spleen and TDLN (data not shown). Interestingly, there was an inverse correlation between foxp3-GFP MFI and CD8:Treg ratio. Mice that had the lowest expression of foxp3-GFP within intra-tumor Tregs had the greatest corresponding CD8:Treg ratio, while those with normal expression had ratios similar to those seen in IgG-treated mice (Figure 5C).

**Figure 6:**
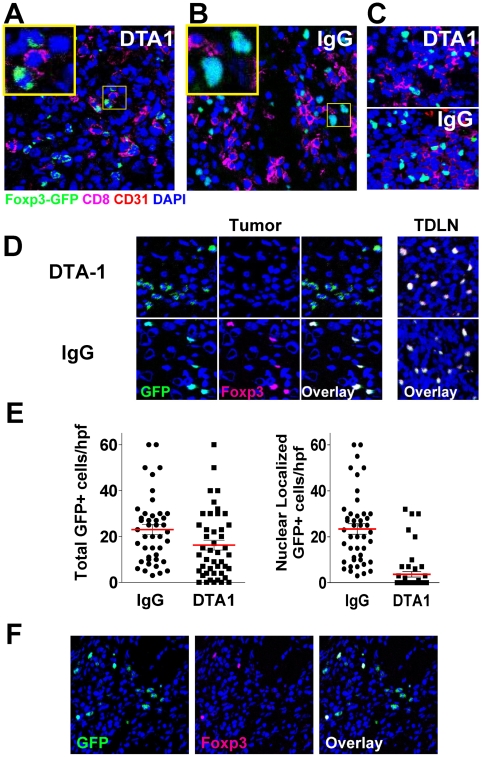
DTA-1-treated mice have abnormal intra-tumor Tregs which have lost foxp3 expression. Tumors were harvested from day 10 B16-bearing *foxp3* ^GFP^ mice (n = 4/group) treated with DTA-1 or IgG on day 4. Tumor sections were stained with anti-CD8 (magenta), anti-CD31 (red, to visualize endothelium), and DAPI (blue, for nuclear staining) and analyzed by immunofluorescence. **A**. DTA-1-treated tumor showing Tregs with irregular borders, weaker foxp3/GFP+ signal, and non-nuclear GFP localization (inset). **B**. IgG-treated tumor showing Tregs with foxp3 protein (GFP+, green) co-localizing with nucleus. **C**. DTA-1- and IgG-treated TDLN demonstrating intact foxp3+ cells. **D**. Left, top: DTA1-treated tumor co-stained with anti-foxp3. Note lack of foxp3 co-staining in cells with abnormal cytoplasmic GFP signal, compared with overlapping nuclear foxp3 and GFP in IgG-treated intra-tumor Tregs (left, bottom) or DTA-1- or IgG-treated TDLN (right). Scale for all images is show in **A** (bar = 25 µm). **E**. The number of GFP+ cells per high-powered field (hpf) were counted, regardless of either intensity or localization (“total”, left graph) or only with bright nuclear GFP signal (i.e. co-localizing with DAPI) (“nuclear GFP,” right graph). Pooled data from a total of 46 (IgG) or 48 (DTA-1) examined hpf (10–12 hpf per tumor ×4 tumors/group) are shown. **F**. Although the majority of DTA-1 treated Tregs have an “abnormal” GFP+ foxp3- phenotype, “intact” and “abnormal” Tregs can be found together within a minority of DTA-1 treated tumor sections.

Therefore, to confirm these findings and further characterize tumor-infiltrating Tregs, we performed immunoflourescent histological analysis of B16 tumors in foxp3*^GFP^* mice. Mice were treated with DTA-1 on day 4 of tumor growth and tumors and TDLN were isolated on day 10, when Treg tumor infiltration in untreated mice begins to peak (Schaer DA, unpublished results). At this time, the total number of GFP+ cells within the DTA-1 treated tumors showed a ∼28% decrease compared to IgG control treated mice (mean 17.8 per high power field (hpf) for DTA-1 vs. 25/hpf for IgG, p = 0.06, Figure 6E). While this decrease was not as dramatic as what was seen with transferred Tregs (Figure 4A), it was on par with the reduced number of Tregs seen by FACS (Figure 3A). More striking, however, was the finding that over 80% of Tregs in DTA-1 treated tumors had an irregular appearance, with weak and aberrant GFP expression that did not co-localize with nuclear DAPI staining (Figure 6A, insert). This is in contrast to IgG tumors where Tregs displayed “normal” overlay of foxp3^GFP^ transcription factor with nuclear DAPI staining. The abnormal phenotype was noted only in DTA-1-treated tumors and not IgG-treated tumors (Figure 6B) or DTA-1-treated TDLN (Figure 6C), demonstrating a specific effect within the tumor microenvironment. Thus, when we quantified the numbers of “normal” Tregs (i.e. co-localizing GFP and nuclear DAPI), we observed a marked decrease within DTA-1-treated tumors (mean 4/hpf) compared with IgG-treated controls (25/hpf, p<0.0001, Figure 6E). This represents a ∼84% reduction in the numbers of “normal” Tregs. Recent reports have demonstrated the plasticity of Tregs and their ability to lose foxp3 expression under inflammatory conditions [25], [26]. Therefore, we asked if the discrepancy in our data was due to loss of foxp3 in the irregular Tregs. Co-staining for foxp3 demonstrated that these aberrant cells were all foxp3-negative (Figure 6D), indicating that they had lost expression of the foxp3 protein. While the irregular Tregs still maintained some GFP protein, it now was no longer linked to foxp3 and its nuclear localization signal. The majority of DTA-1-treated tumor sections contained only the aberrant Tregs; however, some sections contained both normal (foxp3+) and aberrant (foxp3-) GFP+ cells (Figure 6F), demonstrating that this was not a staining artifact.

**Figure 7:**
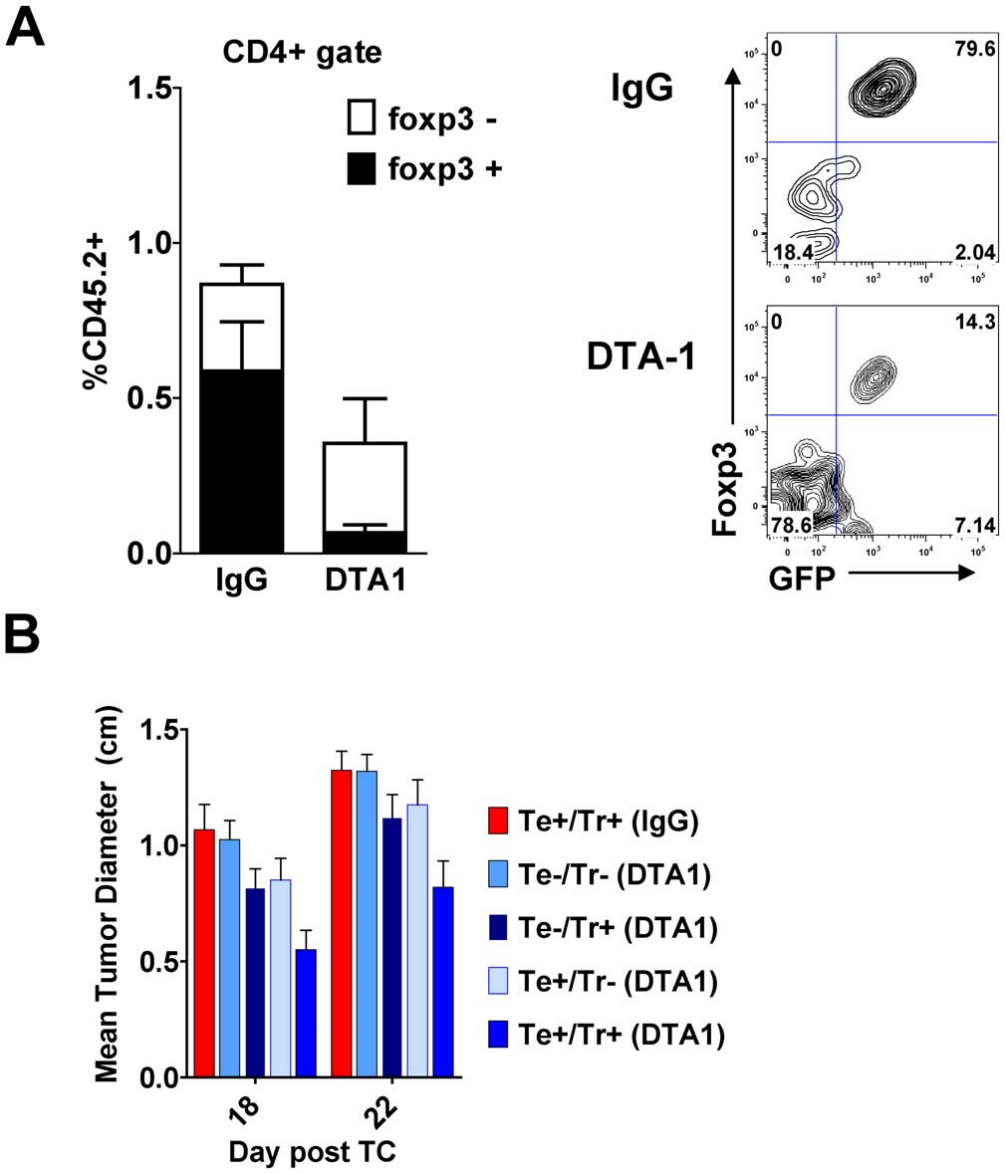
DTA-1-induces foxp3 loss in intra-tumor Tregs after transfer, and the effects of DTA-1 require GITR expression on effector and regulatory T cells. **A**. CD4+GFP+ Tregs isolated from spleens and TDLN of B16-bearing CD45.2+ foxp3*^gfp^* donor mice on day 8 after tumor challenge were transferred (0.7×10^6^ per recipient) into B16-bearing CD45.1+ mice treated with cyclophosphamide and DTA-1 or IgG, as described in Figure 4. Tumor-infiltrating lymphocytes were isolated 2–3 days later and CD45.2+ donor T cells were assessed for their level of foxp3 and GFP expression by FACS. **Left**: The % + SEM of donor (CD45.2+) Tregs found within the total live CD4+ gate is depicted, along with relative proportions that became foxp3-negative. Donor Tregs within DTA-1-treated tumors showed a significantly greater reduction in foxp3 expression (p = 0.001) **Right**:. Representative FACS plots gated on live CD45.2+CD4+ TIL. Data are pooled (n = 7 per group) from 2 independent experiments. **B** RAG1^−/−^ mice (n = 5−9/group) were reconstituted with indicated combinations of effector (Te: CD8+ and CD4+CD25-) and regulatory (Tr: CD4+CD25+) T cells from GITR^−/−^ (−) or GITR^+/+^ (+) littermates (See Figure S5 for schema). After 4 weeks, mice were challenged with B16 and treated with DTA-1 or IgG on day 4. At 18 and 22 days post-challenge, when all tumor-free survival is lost in WT untreated animals (Figure 1A), mean diameter of the Te+/Tr+ (DTA1) group was significantly different from all other groups. (p<0.05; two-tailed student's t-test). Data are pooled from 2 independent experiments.

In order to determine the relative contributions of impaired tumor infiltration and loss of foxp3 expression to the decreased intra-tumor accumulation of Tregs, we repeated the adoptive transfer experiments described in Figure 4 using highly purified (>99% CD4+GFP+, 93% foxp3+ (data not shown)), FACS-sorted donor Tregs from foxp3*^GFP^* mice, in an attempt to track Tregs that become foxp3-negative within the tumor. By gating on donor cells using a congenic marker and assessing dual expression of foxp3 and GFP, we found both decreased Treg infiltration and loss of foxp3 expression within the tumors of DTA-1-treated recipients (Figure 7A), implying that both of these mechanisms are playing a role in this phenomenon. Interestingly, a significant minority of CD4+GFP+ donor cells entering control IgG-treated tumors also became foxp3-negative (and GFP-negative), as described previously for Tregs transferred into lymphopenic hosts [27], [28], which is likely related to the pre-treatment of recipients with cyclophosphamide 1 day prior to transfer. This population, however, was significantly increased following in vivo GITR ligation (mean % of foxp3+ donors becoming foxp3-  = 78% for DTA-1 vs. 34% for IgG, p = 0.001, Figure 7A), demonstrating an additional effect of GITR ligation. We were unable, at least by FACS analysis, to identify the aberrant or “transitioning” GFP+foxp3- cells in these adoptive transfer experiments. As Tregs require continued nuclear foxp3 expression to remain suppressive [29], it is possible that these foxp3-,“former” Tregs have lost regulatory function. Thus, in addition to impairing tumor infiltration, a mechanism by which GITR ligation alters the Teff:Treg balance within the tumor may be alteration of the lineage stability of intra-tumor Tregs.

### Maximal anti-tumor effect of agonist anti-GITR mAb requires GITR expression by both effector and regulatory T cells

Finally, because of these myriad effects on intra-tumor Tregs, as well as current (Figure 3C) and prior [15], [16], [17] data showing enhancement of Teff function, we attempted to confirm that expression of GITR on both Tregs and Teffs was necessary for the protective effect of DTA-1. Therefore, we reconstituted RAG1^−/−^ mice with different combinations of Teffs and Tregs isolated from either GITR^−/−^ or GITR^+/+^ littermates, challenged with B16, and treated with DTA-1 or IgG on day 4 of tumor growth (Figure S5). Although we did not see full protection with DTA-1 in this RAG1^−/−^ reconstitution system, there was a statistically significant delay in tumor growth seen only when both Teff and Treg were GITR +/+ (Figure 7B). Lack of GITR on either population led to an attenuated effect, while lack of GITR on both populations abrogated any protective effect. Taken together, these findings provide further evidence that agonist anti-GITR mAb is modulating the activity of both Teffs and Tregs to effect tumor immunity.

## Discussion

Although prior studies have demonstrated that in vivo GITR ligation results in anti-tumor effects, the exact mechanisms involved remain elusive. This study sought to determine whether GITR ligation on Teffs, Tregs, or both was primarily responsible for these anti-tumor effects, and explore how these subsets were modulated in vivo following GITR ligation. Similar to a study using a fibrosarcoma model [15], we found that GITR ligation was more effective after several days of B16 melanoma growth compared with day 0. This suggested a requirement for initial priming of the immune response in order to induce upregulation of GITR on activated cells. The need for activation before effective GITR ligation is also consistent with our prior study combining DTA-1 with xenogeneic DNA vaccination, in which DTA-1 augmented CD8+ responses and tumor protection only when administered with the second vaccination, and was ineffective when given prior to the initial priming vaccine.[16]

During optimal therapy with DTA-1, it became apparent that Treg tumor infiltration was dramatically decreased compared to untreated animals. This resulted in an enhanced intra-tumor CD8:Treg ratio, without significant changes in the spleen or TDLN. A similar skewed ratio has recently been reported with an agonist anti-OX40 mAb, as well as antagonist anti-CTLA4 mAb, suggesting that an altered intra-tumor Teff:Treg ratio may be predictive of anti-tumor activity of these immune-modulating approaches[22], [23], [30], [31]. Regardless of its prognostic implication, the reduced Treg infiltration without major changes in total CD8+ T cell numbers was our initial evidence that a Treg-intrinsic effect was occurring during DTA-1 treatment.

In exploring the effects of DTA-1 on Tregs further, we discovered that the altered CD8:Treg ratio following GITR ligation was due both to decreased Treg tumor infiltration and to loss of foxp3 within Tregs that did infiltrate the tumor. Transferring tumor-experienced Tregs into DTA-1-treated, tumor-bearing hosts showed that this effect was dependent on Treg GITR expression, as GITR^−/−^ Tregs did not show significant changes in tumor infiltration. Regulation of Treg trafficking to peripheral sites is complex, with a host of different integrins and chemokine receptors being implicated [32], [33]. CD103 (α_E_), CCR7, and CD62L (L-selectin) are involved in Treg trafficking from lymph nodes to inflamed sites, with CD103+CCR7-CD62L- Tregs most capable of homing to and suppressing peripheral inflammation [34], [35]. We did not see significant changes in CD103, CCR7 or CD62L expression on TDLN Tregs 48 or 72 hours following in vivo DTA-1 treatment (Figure S6). In addition, the chemokine receptor CCR4 (specific for CCL17 and CCL22) has been specifically implicated in Treg migration into tumors [36], [37], and we are currently examining alterations in the levels of this and other chemokine receptors on Tregs following GITR ligation in vitro and in vivo.

By histology we observed that most Tregs that do enter the tumor lose foxp3 expression, with some residual GFP expression now localized to the cytosol. In fact, repeating the transfer experiment with a highly-purified Treg population from foxp3*^GFP^* mice confirmed the DTA-1-induced intra-tumor foxp3 loss in this transferred population as well. Whether this loss of foxp3 protein is due to decreased expression, increased degradation, or some combination of the above is not clear. The plasticity of both naturally-arising and induced CD4+foxp3+ Tregs has recently been documented in several studies, with loss of foxp3 expression and suppressive function occurring during conditions of lymphopenia or inflammation/autoimmunity, in some cases even accompanied by gain of effector function [25], [26], [27], [28], [38]. Treg lineage stability may be regulated in part through the integration of external stimuli which either promote/maintain (e.g. IL-2, IL-10, TGF-β) or decrease (e.g. IL-6) foxp3 expression, through modulation of signaling pathways and altered methylation of the foxp3 locus [39], [40]. Interestingly, Luo et al recently reported that TGF-β1-mediated induction of foxp3 in CD4+CD25- cells was due to inhibition of phospho-ERK and down-regulation of DNA methyltransferases, leading to demethylation of the foxp3 promoter [41]. Signaling through GITR is known to activate the MAP kinase pathway and induce ERK phosphorylation [4], [42], [43]. Thus, GITR ligation may antagonize the effects of TGF-β on pERK and inhibit downstream signaling which normally induces foxp3 gene expression. In support of this, we have found that in vitro GITR ligation is able to block TGF-β-mediated up-regulation of foxp3 in activated CD4+foxp3- T cells (Figure S7), suggesting an effect on gene expression. Whether this mechanism, likely in concert with other inflammatory stimuli present within the tumor microenvironment, plays a role in the selective loss of foxp3 observed in tumor-infiltrating Tregs is currently under investigation.

Despite this effect in the tumor, DTA-1 did not modulate the ex vivo suppressive capacity of Tregs isolated from TDLN, consistent with prior ex vivo [17], [18] and in vitro [6], [44] studies. In all of these studies, Treg suppressive ability was maintained during GITR ligation, and collectively these data refute the argument that GITR ligation globally makes Tregs unable to suppress. Still, it remains possible that intra-tumor Tregs, which lose foxp3 expression, have diminished suppressive capacity. This possibility is supported by recent data demonstrating that Tregs require constant foxp3 expression for the continued expression of suppressive genes, and for the repression of inflammatory genes as well.[29] Unfortunately, we were unable to test the ex vivo suppressive capacity of these “former” Tregs, as the yield of purified live GFP+ TIL isolated from DTA-1-treated, B16-bearing *foxp3* ^GFP^ mice was always too low after FACS-sorting (data not shown). Thus, this question remains open for now.

Somewhat surprisingly, we did not find that in vivo GITR ligation in our B16 model made CD8+ Teffs from TDLN resistant to Treg-mediated suppression ex vivo, in contrast to prior observations with CD4+ [6], [17] or CD8+ Teffs [19]. One explanation for this discrepancy may be that we isolated CD8+ T cells from the TDLN 72 hours after DTA-1. Since CD8+ T cells are less responsive in general to GITR signaling than CD4+ T cells [3], [6], [10], [11], it is possible that the level of GITR signaling required for “resistance” to Tregs had waned by the time these cells were activated ex vivo. In addition, we used bulk CD8+ Teffs isolated from TDLN, only a minority of which were likely activated by tumor antigens and therefore amenable to co-stimulation by DTA-1. In contrast, the study by Nishikawa et al demonstrating generation of “Treg-resistant” CD8+ cells used activated, tetramer-purified, antigen-specific CD8+ T cells in their suppression assays.[19] Based on these data, it is possible that a population of activated, tumor-specific CD8+ T cells from DTA-1-treated B16-bearing TDLN might be similarly “Treg-resistant.” While we did not demonstrate that GITR ligation directly modulated suppression or resistance to suppression, we did observe a reproducible increase in the intra-tumor effector function of tumor specific pmel-1 T cells. Therefore, even if Tregs still retain suppressive capacity following GITR ligation, their relative numerical disadvantage in the tumor may render them less effective overall.

Considering our evidence that DTA-1 directly modulated Tregs as well as Teffs, we reconstituted RAG1^−/−^ mice with GITR^−/−^ or GITR^+/+^ Teffs and Tregs, to confirm which cell types were able to respond to GITR ligation. While the anti-tumor effect of DTA-1 in this system was modest, the delay in tumor growth was reduced if either Tregs or Teffs lacked GITR, and was non-existent if both subsets were GITR-negative. It is possible that the weakened effect of DTA-1 was due to inadequate peripheral reconstitution. Alternatively, there could be a contributing role for GITR ligation on B cells or NK-T cells, both of which were absent in our RAG1^−/−^ mice and have been reported recently to have augmented activation with GITR co-stimulation.[17], [45]. Regardless, these data suggest that GITR must be present on both Treg and Teff populations for the full anti-tumor effects of DTA-1.

In conclusion, a single dose of agonist anti-GITR mAb can induce T-cell-mediated rejection of an aggressive, poorly immunogenic tumor. Additionally, our data supports the idea that DTA-1 modulates both Teffs and Tregs during therapy. GITR ligation on Tregs appears to cause impaired accumulation within the tumor through Treg lineage instability and a block of trafficking. This leads to greater intra-tumor Teff:Treg ratios and more potent Teff activity. Furthermore, those Tregs which do traffic to the tumor are likely limited in their suppressive capacity as denoted by loss of continued nuclear foxp3 protein expression. Importantly, the preservation of global Treg function and limitation of this skewed ratio to the tumor may make widespread loss of tolerance and autoimmune consequences with in vivo GITR ligation less likely. These data provide further support for the development of this strategy as a therapeutic approach to human cancer.

## Materials and Methods

### Ethics Statement

Mice were maintained according to NIH Animal Care guidelines, under a protocol 96-04-017 approved by the MSKCC Institutional Animal Care Committee.

### Mice

C57BL/6 Thy1.2+ and Thy1.1+ mice were obtained from Jackson Laboratory (Bar Harbor, ME). Thy1.1+ pmel-1 T-cell receptor transgenic mice have been reported.[46] *foxp3* ^GFP^ knock-in mice were a gift from Dr. A. Rudensky (MSKCC, NY, NY).[20] GITR^−/−^ and GITR^+/+^ littermates (Sv129 x C57BL/6 background)[47] were a gift from Dr. P.P. Pandolfi (MSKCC, NY, NY) and were backcrossed >10 generations onto C57BL/6 background using a speed congenic system[48].

### Cell lines and tumor challenge

B16F10/LM3 (hereafter called B16) is derived from the B16F10 line provided by I. Fidler (M.D. Anderson Cancer Center, Houston, TX). EL4, (American Type Culture Collection, ATCC Number: TIB-39, Manassas, VA), C57BL/6 lymphoma cell line was used as an antigen-presenting cell in intracellular cytokine assays. Cell lines were cultured as described.[49] For tumor-free survival experiments, 50,000 B16 cells were injected intradermally in serum-free RPMI media, and tumor diameters were measured with calipers every 2–3 days. For experiments requiring recovery of tumor-infiltrating lymphocytes (TIL), 500,000 B16 cells in growth factor-reduced, phenol red-free Matrigel (BD Biosciences) were injected subcutaneously.

### Lymphocyte isolation

Spleens, tumor-draining inguinal lymph nodes, and tumors were homogenized through 0.22 µm strainers to produce single cell suspensions. RBCs were lysed from spleens using an ammonium chloride lysis buffer. TILs were isolated from tumor suspensions by density gradient centrifugation using Percoll (GE Healthcare, Piscataway, NJ). Briefly, cell pellets were suspended in 80% Percoll, overlayed with 40% Percoll, and centrifuged at 2000×g for 30 minutes. Cells at the interface were collected, washed, and used for FACS or functional assays. Intracellular cytokine assays for expression of IFN-γ and CD107a following peptide restimulation were performed as reported.[16]

Isolation of CD8+, CD4+foxp3+, and CD4+foxp3- cells from foxp3*^GFP^* mice was performed on a Cytomation MoFlo cell sorter. Isolation of CD8+, CD4+CD25+, and CD4+CD25- cells from other mouse strains was performed using MACS microbead separation kits (Miltenyi Biotec Inc., Auburn, CA). pmel-1 CD8+ cells were labeled with CFSE 2.5 µM prior to adoptive transfer. Transferred cells were injected intravenously by tail vein in 200 µl sterile PBS. For some adoptive transfer experiments, recipients were pre-treated with cyclophosphamide (Sigma-Aldrich) 250 mg/kg in 500 µl sterile PBS intraperitoneally one day prior to transfer.

### Antibodies and FACS analysis

The DTA-1 hybridoma, from S. Sakaguchi (Kyoto University, Kyoto, Japan), and the OX86 hybridoma, from A. Weinberg (Earle Chiles Research Institute, Portland, OR), were used to produce agonist anti-GITR and anti-OX40 mAb, respectively, by the MSKCC Monoclonal Antibody Core Facility. One mg affinity-purified DTA-1 in 500 µL PBS was injected intraperitoneally. Purified Rat IgG (Sigma-Aldrich) was used as a control. Foxp3 staining was performed using the Mouse Regulatory T-Cell Staining Kit (eBioscience, San Diego, CA). Other flow cytometry antibodies were from BD Biosciences (San Jose, CA). hgp100_25-33_-D^b^-tetramer, containing the D^b^ epitope KVPRNQDWL, has been described.[16] For fixed/permeablized samples, LIVE/DEAD® Fixable Dead Cell Stain Kit (Invitrogen, Carsbad, CA) was used to identify dead cells; for other samples DAPI was used. Samples were run on a BD 4-color FACSCalibur or 12-color LSRII cytometer, and were analyzed using FlowJo (Treestar, San Carlos, CA).

### Immunofluorescence

B16-matrigel tumor samples were harvested on day 10 and fresh frozen tissue was embedded in OCT. Sequential 5 µm sections were collected and stained with anti-CD31 biotin (BD Pharmingen), anti-CD8 Alexa fluor 657 (Caltag/Invitrogen), anti-foxp3 APC (ebiosicence), anti-Caspase 3 (BD Pharmingen) and DAPI for nuclear stain. TUNEL staining (TdT and biotin-dUTP from Roche Diagnostics Corp.) was performed after proteinase K digestion (Sigma) and detected using Alexa-633 Avidin (Molecular Probes). Images were acquired with a Leica upright confocal microscope using 20X objective at 2048×2048 resolution. Acquisition was performed using IgG-treated LN sections to set PMT detector settings at non-saturating conditions, with background levels set using isotype control or secondary antibody alone. Detector voltages were then maintained for each staining condition across all sections.

### Treg suppression assay

10^5^ CD8+ Teff were labeled with 1.25 µM CFSE and cultured with CD4+foxp3+ cells (FACS-sorted from *foxp3* ^GFP^ mice) or CD4+CD25+ cells (MACS-selected from C57BL/6 mice) at indicated ratios, along with 10^5^ T-cell-depleted, irradiated (444 cGy) splenocytes and 1 µg/ml anti-CD3 mAb. After 4 days, cells were harvested, stained with anti-CD8 and DAPI and analyzed by FACS.

### Quantitative real time PCR

Total RNA was extracted from FACS-sorted Tregs using RNeasy Micro Kit (Qiagen, CA) and cDNA synthesized using High Capacity cDNA Reverse Transcription Kit (Applied Biosystems, CA) according to manufacturer's instructions. All primers and probes were from TaqMan® Gene Expression Assays (Applied Biosystems). Real-time PCR reactions were prepared with 4 ul cDNA (corresponding to 30 ng of RNA), 1.25 ul of 20× Primer/TaqMan probe mixture, 12.5 ul of 2×TaqMan Universal PCR Master Mix and 7.25 ul of DNase/Rnase-free water. All amplifications were done using ABI 7500 Real Time PCR system. Each gene was amplified in triplicate and cDNA concentration differences were normalized to GAPDH. Relative gene expression changes of the target genes in DTA-1-treated group compared to IgG-treated group were calculated by 2-ddCt method (25) using average Ct (threshold cycle) of triplicates from the IgG-treated group as a calibrator. Fold increase or decrease in gene expression was calculated and the values were log10-transformed before plotting.

### Statistical analysis

Differences in long-term tumor-free survival were evaluated by log rank analysis of Kaplan-Meier survival curves (GraphPad Prism 4.0). Statistical differences between groups were determined by analyzing means + standard errors of replicate mice or wells by two-tailed Student's *t* test (GraphPad Prism 4.0).

## Supporting Information

Figure S1 DTA-1 induces rejection of B16 melanoma most effectively at day 4. C57BL/6 mice were challenged with 50,000 B16 cells intradermally and treated with DTA-1 or Rat IgG on indicated days. Pooled data from 5 experiments (n = 30−50 total mice/group). Mice alive at day 60 without tumor were considered long-term tumor-free survivors.
(0.14 MB TIF)

Figure S2 GITR ligation does not decrease Treg expression of Granzyme B, IL10, or TGF-β. For in vitro analysis, purified CD4+foxp3+ cells (from spleens + LN of naïve foxp3GFP mice) were treated with anti-CD3 mAb 1 µg/ml, anti-CD28 mAb 1 µg/ml, and DTA-1 or Rat IgG 10 µg/ml for 48 hours, followed by RNA extraction and quantitative real-time PCR as per Supplemental Methods below. For in vivo analysis, RNA was extracted from CD4+foxp3+ cells purified from TDLN of day 10 B16-bearing foxp3GFP mice treated 3 days earlier with DTA-1 or IgG 1 mg i.p. The relative change (on log10 scale) in gene expression (normalized to GAPDH) for DTA-1-treated Tregs compared to IgG-treated Tregs is depicted.
(0.13 MB TIF)

Figure S3 No changes seen in absolute number of CD8 T cells/gram of tumor after DTA-1 treatment. Representative counts from 3 independent experiments showing numbers of T cells per gram of tumor. CD8 T cells are gated on CD45+ CD8+ and Tregs are gated on CD45+CD4+,foxp3+ inside the live gate from tumors, 10 days after B16 inoculation,(6 days post DTA-1 treatment).
(0.19 MB TIF)

Figure S4 Abnormal GFP+ Tregs within DTA-1-treated tumors are not undergoing apoptosis. Day 10 B16-matrigel tumors from foxp3GFP mice treated with 1 mg DTA-1 or IgG on day 4 were harvested and processed for TUNEL staining (A) or flow cytometry (B) as per Methods. A) Representative images show lack of TUNEL positive staining of irregular Tregs (arrows) inside DTA1 treated tumors. B) Representative staining of live (DAPI-) tumor-infiltrating Tregs (CD4+GFP+) show no difference in frequency of apoptotic (Annexin V+) cells between DTA-1 and IgG-treated tumors. Gate placement based on fluorescence intensity of stained cells without addition of Annexin V.
(3.93 MB TIF)

Figure S5 Schema for reconstitution of RAG1−/− mice with GITR−/− or GITR+/+ effector (Teff) or regulatory (Treg) T cells.
(0.18 MB TIF)

Figure S6 In vivo GITR ligation does not alter surface expression of CD103, CD62L, or CCR7 on Tregs within tumor-draining lymph nodes. B16-bearing C57BL/6 mice were treated with DTA-1 or IgG 1 mg on day 4 and tumor-draining lymph nodes harvested 48 hours later. Isolated lymphocytes were stained for FACS. Expression of indicated molecules on gated live CD4+foxp3+ Tregs from representative mice are depicted. Similar findings were observed 72 hours after DTA-1 or IgG treatment (data not shown).
(0.30 MB TIF)

Figure S7 GITR ligation blocks TGF-β-mediated in vitro peripheral conversion to induced Tregs. 5×104 CD4+foxp3- cells (FACS-sorted from naive foxp3GFP splenocytes) were cultured for 5 days at 37°C with 1.5×105 irradiated T-cell depleted splenocytes, 0.1 µg/ml anti-CD3 mAb, 1 µg/ml anti-CD28 mAb, and indicated concentrations of IgG, DTA-1, or OX86 (agonist anti-OX40 mAb). 40 U IL-2 and 5 ng/ml TGF-β1 was added to each well after 48 hours in culture. After a total of 5 days incubation, cells were harvested, stained with anti-CD4 and DAPI and analyzed by FACS. The % foxp3+GFP+ from gated CD4+DAPI- are shown. Similar findings were seen using anti-CD3 mAb at 0.01 or 1 µg/ml (data not shown). Data are from 1 of 3 representative experiments.
(0.43 MB TIF)
