# Supplementary figures and images for: Agonist Anti-GITR Monoclonal Antibody Induces Melanoma Tumor Immunity in Mice by Altering Regulatory T Cell Stability and Intra-Tumor Accumulation
Source: PLoS One. 2010 May 3;5(5):e10436. doi: 10.1371/journal.pone.0010436 (PMC2862699; doi:10.1371/journal.pone.0010436)

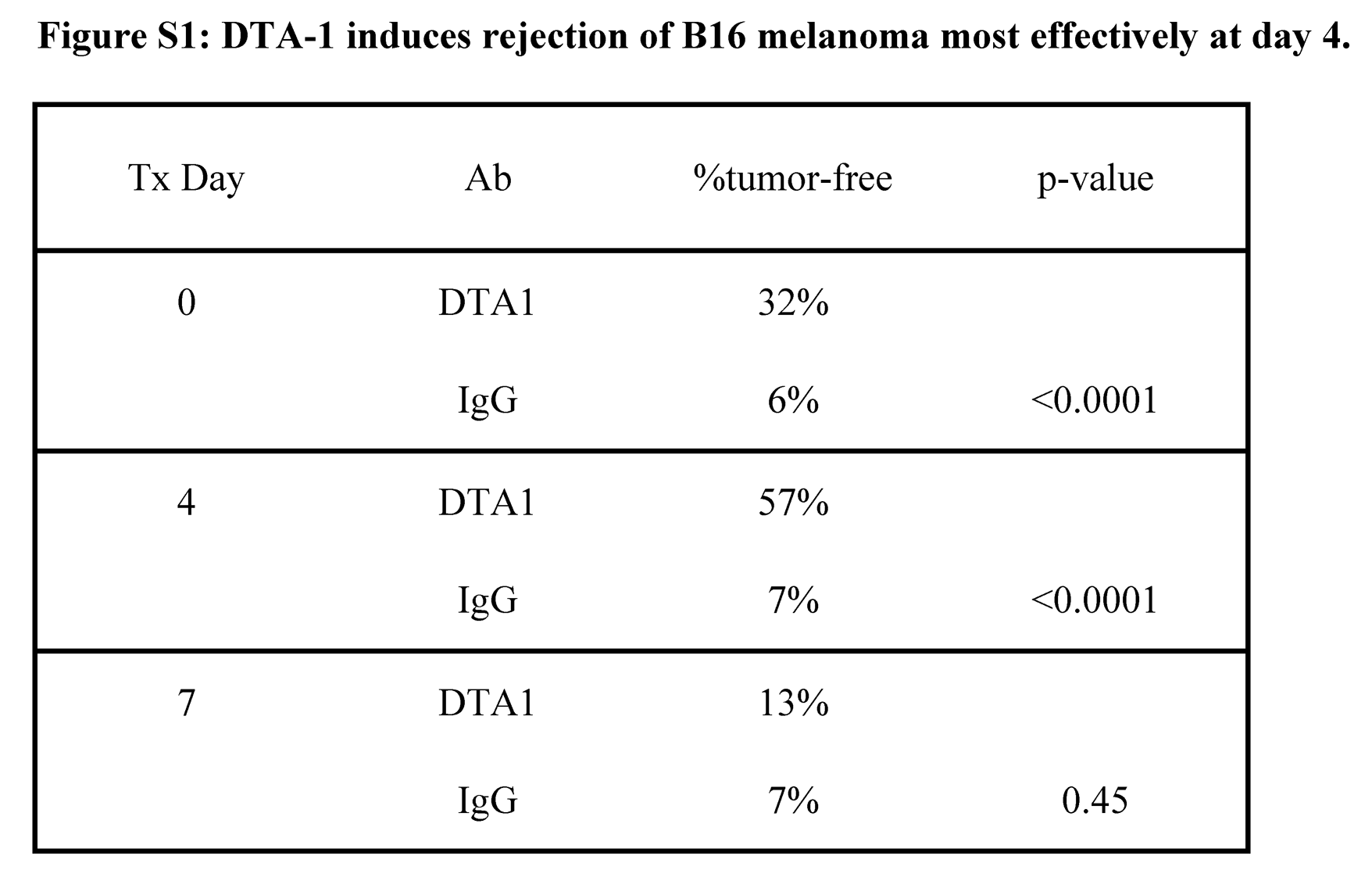

Supplement: Figure S1 — DTA-1 induces rejection of B16 melanoma most effectively at day 4. C57BL/6 mice were challenged with 50,000 B16 cells intradermally and treated with DTA-1 or Rat IgG on indicated days. Pooled data from 5 experiments (n = 30−50 total mice/group). Mice alive at day 60 without tumor were considered long-term tumor-free survivors. (0.14 MB TIF) [file pone.0010436.s001.tif]

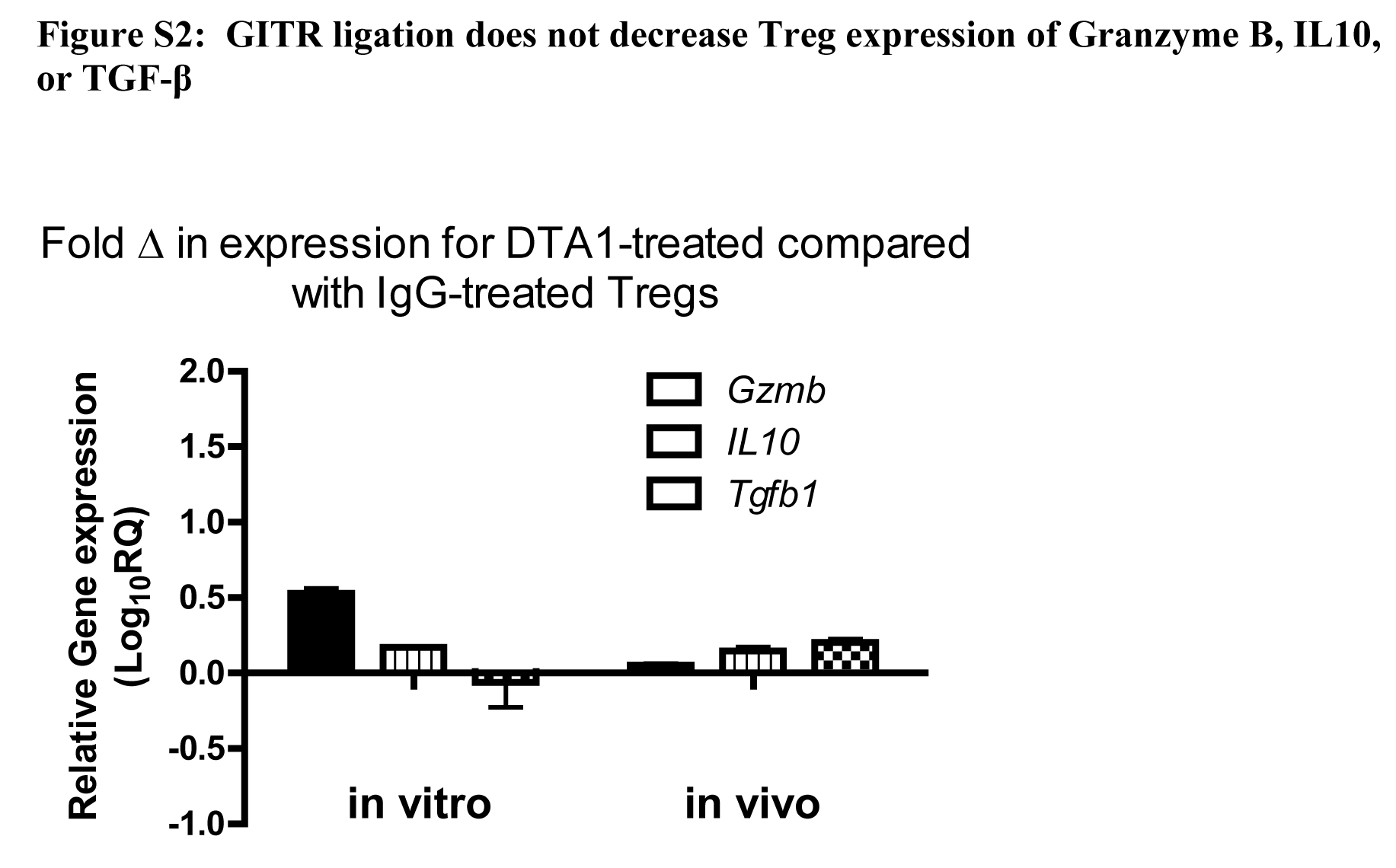

Supplement: Figure S2 — GITR ligation does not decrease Treg expression of Granzyme B, IL10, or TGF-β. For in vitro analysis, purified CD4+foxp3+ cells (from spleens + LN of naïve foxp3GFP mice) were treated with anti-CD3 mAb 1 µg/ml, anti-CD28 mAb 1 µg/ml, and DTA-1 or Rat IgG 10 µg/ml for 48 hours, followed by RNA extraction and quantitative real-time PCR as per Supplemental Methods below. For in vivo analysis, RNA was extracted from CD4+foxp3+ cells purified from TDLN of day 10 B16-bearing foxp3GFP mice treated 3 days earlier with DTA-1 or IgG 1 mg i.p. The relative change (on log10 scale) in gene expression (normalized to GAPDH) for DTA-1-treated Tregs compared to IgG-treated Tregs is depicted. (0.13 MB TIF) [file pone.0010436.s002.tif]

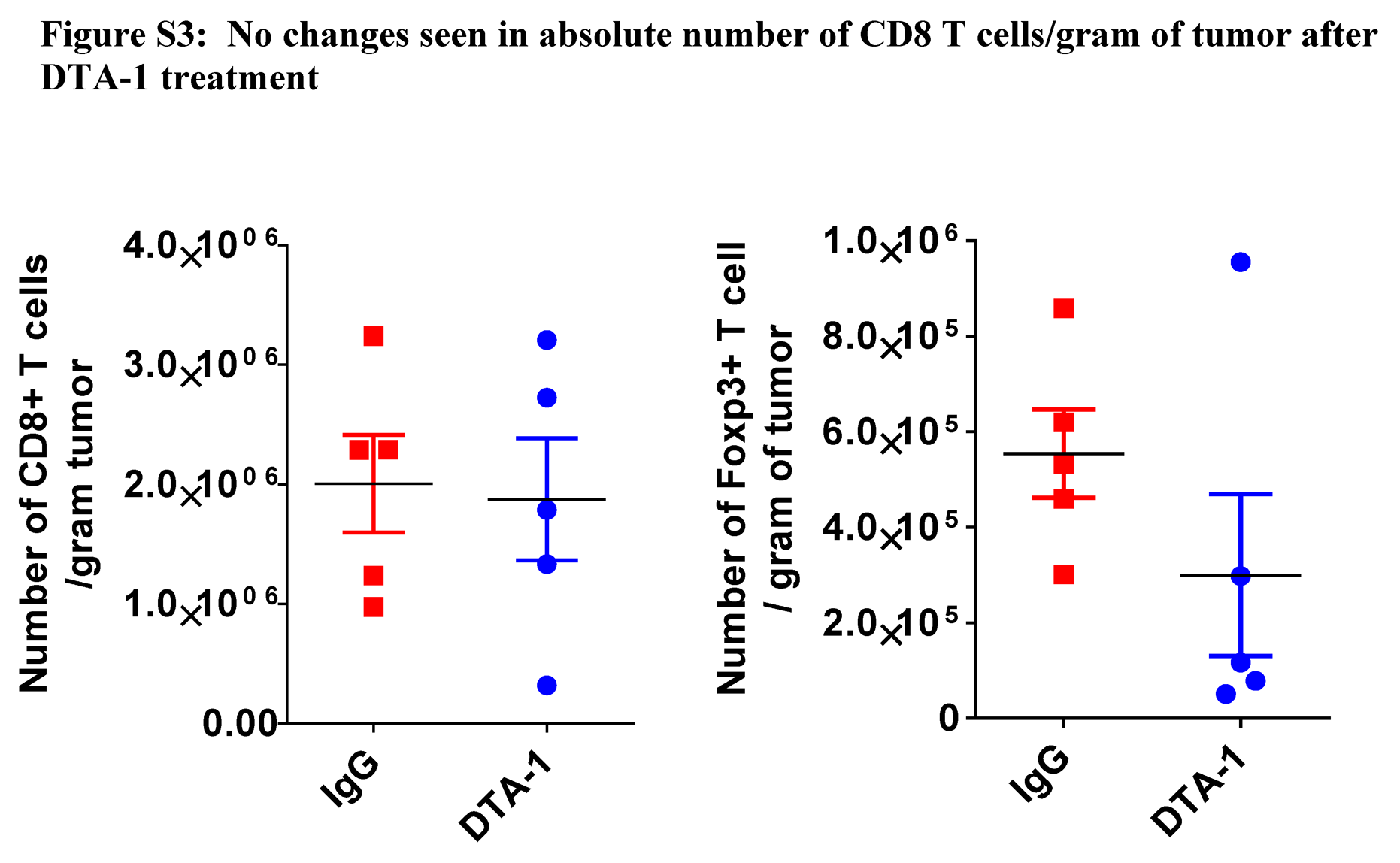

Supplement: Figure S3 — No changes seen in absolute number of CD8 T cells/gram of tumor after DTA-1 treatment. Representative counts from 3 independent experiments showing numbers of T cells per gram of tumor. CD8 T cells are gated on CD45+ CD8+ and Tregs are gated on CD45+CD4+,foxp3+ inside the live gate from tumors, 10 days after B16 inoculation,(6 days post DTA-1 treatment). (0.19 MB TIF) [file pone.0010436.s003.tif]

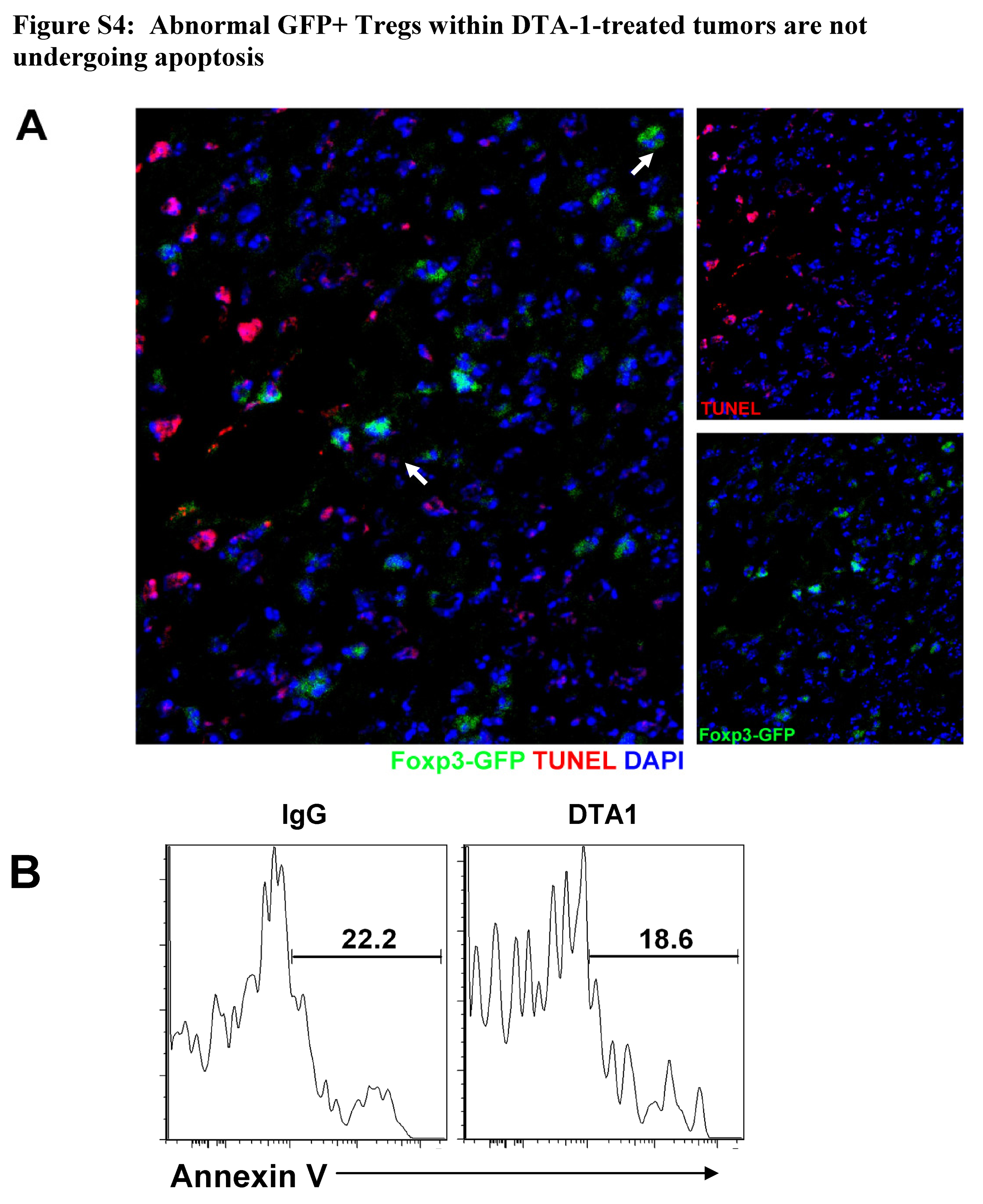

Supplement: Figure S4 — Abnormal GFP+ Tregs within DTA-1-treated tumors are not undergoing apoptosis. Day 10 B16-matrigel tumors from foxp3GFP mice treated with 1 mg DTA-1 or IgG on day 4 were harvested and processed for TUNEL staining (A) or flow cytometry (B) as per Methods. A) Representative images show lack of TUNEL positive staining of irregular Tregs (arrows) inside DTA1 treated tumors. B) Representative staining of live (DAPI-) tumor-infiltrating Tregs (CD4+GFP+) show no difference in frequency of apoptotic (Annexin V+) cells between DTA-1 and IgG-treated tumors. Gate placement based on fluorescence intensity of stained cells without addition of Annexin V. (3.93 MB TIF) [file pone.0010436.s004.tif]

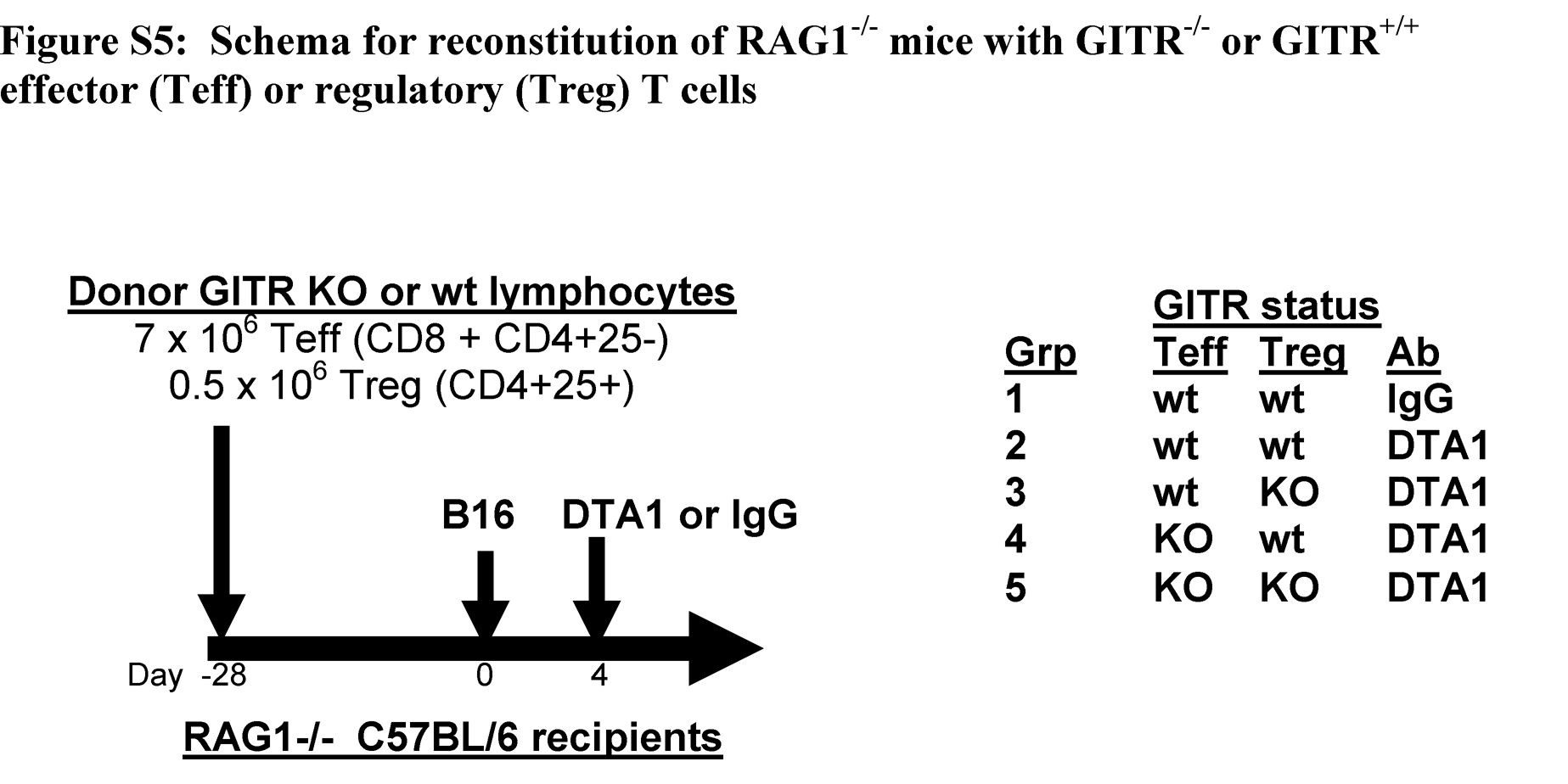

Supplement: Figure S5 — Schema for reconstitution of RAG1−/− mice with GITR−/− or GITR+/+ effector (Teff) or regulatory (Treg) T cells. (0.18 MB TIF) [file pone.0010436.s005.tif]

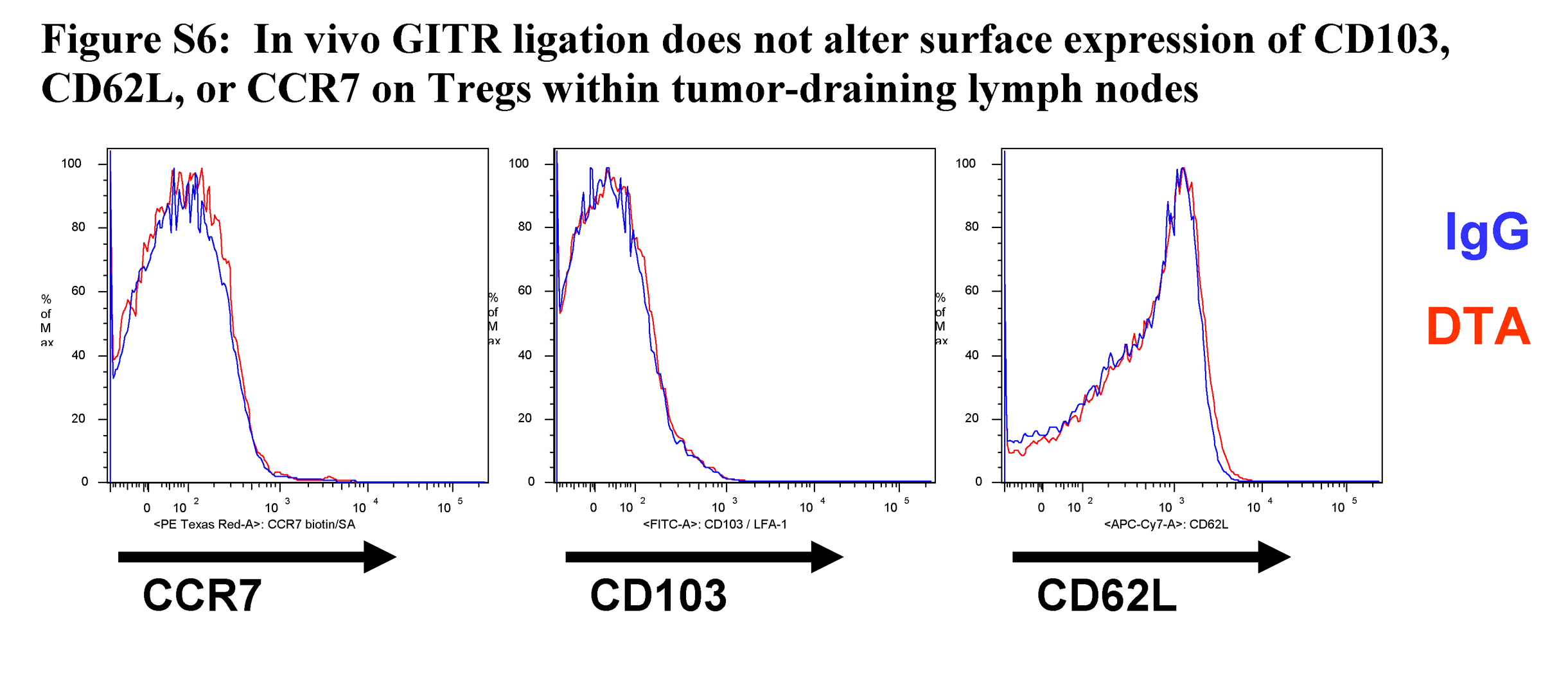

Supplement: Figure S6 — In vivo GITR ligation does not alter surface expression of CD103, CD62L, or CCR7 on Tregs within tumor-draining lymph nodes. B16-bearing C57BL/6 mice were treated with DTA-1 or IgG 1 mg on day 4 and tumor-draining lymph nodes harvested 48 hours later. Isolated lymphocytes were stained for FACS. Expression of indicated molecules on gated live CD4+foxp3+ Tregs from representative mice are depicted. Similar findings were observed 72 hours after DTA-1 or IgG treatment (data not shown). (0.30 MB TIF) [file pone.0010436.s006.tif]

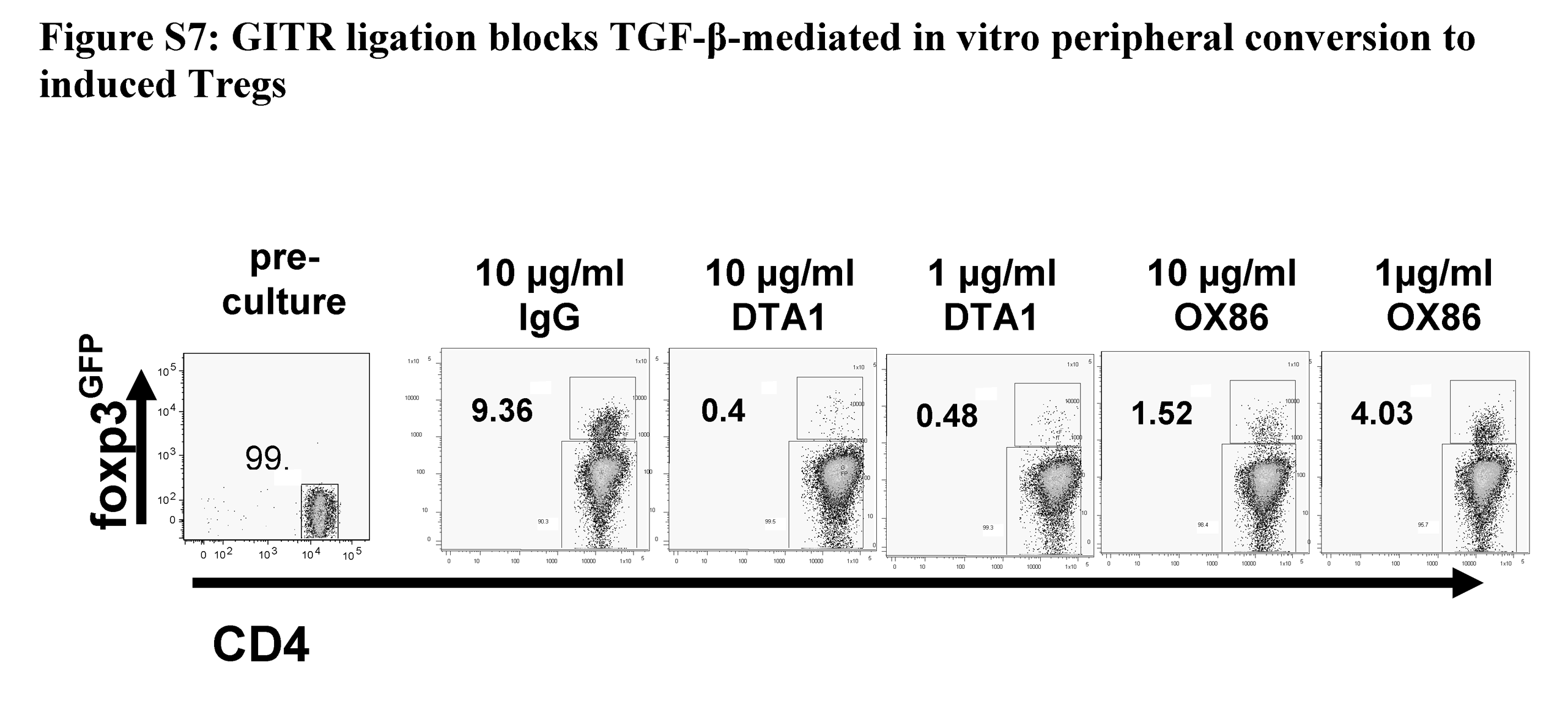

Supplement: Figure S7 — GITR ligation blocks TGF-β-mediated in vitro peripheral conversion to induced Tregs. 5×10^4^ CD4+foxp3- cells (FACS-sorted from naive foxp3GFP splenocytes) were cultured for 5 days at 37°C with 1.5×10^5^ irradiated T-cell depleted splenocytes, 0.1 µg/ml anti-CD3 mAb, 1 µg/ml anti-CD28 mAb, and indicated concentrations of IgG, DTA-1, or OX86 (agonist anti-OX40 mAb). 40 U IL-2 and 5 ng/ml TGF-β1 was added to each well after 48 hours in culture. After a total of 5 days incubation, cells were harvested, stained with anti-CD4 and DAPI and analyzed by FACS. The % foxp3+GFP+ from gated CD4+DAPI- are shown. Similar findings were seen using anti-CD3 mAb at 0.01 or 1 µg/ml (data not shown). Data are from 1 of 3 representative experiments. (0.43 MB TIF) [file pone.0010436.s007.tif]
